# Beyond Weight Loss: Rethinking Dietary and Lifestyle Interventions for Liver Disease Modification in MASLD

**DOI:** 10.3390/nu18193202

**Published:** 2026-09-28

**Authors:** Abdulrahman Almujaidel, Eman Marzouk

**Affiliations:** Department of Public Health, College of Applied Medical Sciences, Qassim University, P.O. Box 6666, Buraydah 51452, Saudi Arabia

**Keywords:** metabolic dysfunction-associated steatotic liver disease, lifestyle intervention, weight loss, liver disease modification, dietary intervention, physical activity, surrogate endpoints, causal inference, target-trial emulation, public health

## Abstract

Metabolic dysfunction-associated steatotic liver disease (MASLD) is commonly managed with dietary and lifestyle intervention, and weight loss is often used as a key marker of treatment success. However, changes in body weight alone do not fully describe the effects of lifestyle intervention on liver disease. This narrative review asks when lifestyle-induced changes can support a claim of liver disease modification. Current guidance generally associates sustained weight loss of at least 5% with reduced liver fat, 7–10% with reduced liver inflammation, and at least 10% with improvement in fibrosis. The review distinguishes weight loss from biological response, pathological improvement, and long-term clinical benefit. It considers how intervention exposure, biological response, outcome type, and duration of follow-up should be interpreted together. It also addresses differences in patient response, long-term adherence, and the limits of different study designs. Randomized trials, long-term observational studies, and target-trial emulation are considered in relation to what they can establish about causality and long-term benefit. We propose a five-question framework for evaluating lifestyle-induced liver disease modification: Who? What? Through which pathway? What outcome? For how long and with what evidence? Weight loss remains an important treatment target, but it should not be considered synonymous with liver disease modification. Claims of disease modification should reflect the level of evidence available. This framework provides a clearer way to distinguish short-term improvement from meaningful and durable modification of MASLD.

## 1. Introduction

Metabolic dysfunction-associated steatotic liver disease (MASLD) is among the most prevalent chronic liver diseases worldwide. Much of the evidence underlying current prevalence estimates comes from studies conducted under the former definition of nonalcoholic fatty liver disease (NAFLD), which reported a global prevalence of approximately 30%. MASLD encompasses a broad spectrum of disease, ranging from isolated steatosis to metabolic dysfunction-associated steatohepatitis (MASH), progressive fibrosis, cirrhosis, and hepatocellular carcinoma [1,2,3].

The clinical significance of MASLD extends beyond hepatic fat accumulation. Progressive fibrosis is closely associated with adverse liver-related outcomes. Together with the broader cardiometabolic burden of MASLD, this underscores the need to prevent disease progression rather than merely reduce steatosis [3,4,5]. Accordingly, lifestyle intervention is central to current MASLD management. Dietary modification, improved diet quality, physical activity, exercise, and weight reduction when appropriate form the basis of treatment [3,4].

The evidence supporting intentional weight loss is substantial. In a prospective study with paired liver biopsies, greater weight loss after 52 weeks of lifestyle intervention was independently associated with improvement in the major histological features of steatohepatitis. Among participants who achieved at least 10% weight loss, 90% had resolution of steatohepatitis, and 45% experienced fibrosis regression [6]. Earlier randomized studies documented histological benefit from intensive lifestyle intervention. In a 48-week trial, the intervention group achieved a mean weight loss of 9.3% and was more likely to show substantial histological improvement than the structured education group [7,8].

These findings reinforce weight reduction as an important therapeutic target in MASLD. Current guidance recommends sustained weight loss of at least 5% to reduce liver fat, 7% to 10% to reduce liver inflammation, and at least 10% to improve fibrosis [3]. Yet these targets do not fully capture the biological response to lifestyle intervention.

Body weight provides an accessible but incomplete measure of biological response. Similar changes in body weight may reflect different changes in body composition and metabolic function, while meaningful hepatic improvement may occur without substantial weight loss. These limitations are discussed further in Section 6.

Exercise also illustrates that liver improvement may occur without substantial weight loss. Randomized trials and meta-analyses indicate that exercise can reduce hepatic steatosis and improve cardiometabolic health, although evidence for histological improvement, fibrosis regression, and long-term liver-related outcomes remains less certain [3,9].

Dietary interventions likewise extend beyond caloric restriction. Current recommendations emphasize diet quality, dietary patterns resembling the Mediterranean diet, lower consumption of ultra-processed foods rich in sugars and saturated fat, and avoidance of sugar-sweetened beverages [3,10]. These approaches may affect hepatic and metabolic pathways beyond changes in body weight, although evidence for long-term clinical liver outcomes remains limited [3].

Lifestyle studies assess outcomes with different clinical significance. Changes in aminotransferases, hepatic fat, liver stiffness, and non-invasive fibrosis markers may indicate biological improvement but do not provide equivalent evidence of histological improvement or prevention of liver-related events. Distinguishing short-term surrogate responses from durable changes in disease progression and clinical outcomes is therefore central to defining liver disease modification [3,4,11].

Evidence becomes less direct for major clinical outcomes, and current European guidance calls for longer follow-up to determine whether lifestyle-induced weight loss reduces liver-related events and mortality [3]. Sustaining lifestyle change in routine care is an additional challenge. The Treatment of NAFLD in a Real-World Setting (TARGET-NASH) observational cohort highlighted the difference between achieving initial weight loss and maintaining it over time. This distinction is particularly important when evaluating a chronic and potentially progressive disease [12].

Treatment response is heterogeneous, as differences in disease stage, metabolic status, adiposity distribution, physical fitness, and ability to sustain lifestyle changes may lead to different hepatic outcomes despite similar interventions and weight loss [3,4]. Weight loss may function as a therapeutic target, a pathway through which an intervention exerts benefit, and an accessible but incomplete marker of the overall biological response [3,6,13].

This narrative review examines dietary and lifestyle interventions in MASLD and their role in modifying liver disease. It focuses on weight loss, diet quality, physical activity, body composition, the gut–liver axis, differences in treatment response, and the importance of meaningful and lasting outcomes. It also considers how randomized trials, long-term studies, and target-trial emulation can help assess lasting liver benefits. The review further examines how these responses should be interpreted in relation to liver-specific outcomes and the evidence required to support claims of liver disease modification.

Weight loss remains a major therapeutic objective in MASLD. However, the success of dietary and lifestyle interventions should ultimately be judged by whether they produce meaningful, durable, and credible evidence of liver disease modification. Figure 1 provides the conceptual framework used throughout this review and will be referred to when interpreting the intervention pathways, biological responses, liver outcomes, and evidence discussed in the following sections.

## 2. Literature Search Strategy

This narrative review was based on a focused search of the literature on lifestyle interventions and liver disease in MASLD. PubMed, Scopus, Web of Science, and Google Scholar were searched. Search terms included combinations related to MASLD, non-alcoholic fatty liver disease, lifestyle intervention, diet, dietary patterns, physical activity, exercise, weight loss, behavioral intervention, liver fat, fibrosis, histological response, clinical outcomes, adherence, sustainability, treatment response, surrogate endpoints, causal inference, and target-trial emulation. The search covered studies published from 2000 to 2026 and was closed on 28 August 2026. Earlier landmark publications were included when relevant.

The search identified 1390 records. After removal of 392 duplicates, 998 records were screened, of which 790 were excluded. A total of 208 full-text publications were assessed for eligibility, and 156 were excluded, leaving 52 publications from the database search for the qualitative synthesis. Both authors participated in study selection and assessed the relevance of potentially eligible publications. Additional relevant publications were identified through screening the reference lists of key studies, clinical guidelines, and recent reviews and were included when appropriate.

The review included original research, randomized and non-randomized clinical studies, prospective cohort studies, systematic reviews, meta-analyses, narrative reviews, clinical guidelines, consensus statements, and methodological papers published in English. Publications were selected based on their relevance to the main questions of the review. These included the effects of lifestyle interventions on liver outcomes, the role of weight loss, differences in treatment response, intervention exposure, long-term adherence, interpretation of surrogate and histological outcomes, and the strength of evidence for long-term liver disease modification. Priority was given to publications with direct liver-related outcomes, long-term follow-up, clinically relevant comparisons, or clear relevance to causal interpretation.

Publications were not included when they were unrelated to the aims of the review, focused mainly on non-hepatic outcomes without clear relevance to liver disease, lacked sufficient scientific detail, or were conference abstracts without a full publication. Duplicate reports were removed before screening. As this was a narrative review, formal systematic-review procedures, risk-of-bias assessment, and quantitative meta-analysis were not performed. The evidence was critically synthesized to examine when lifestyle-induced changes may support meaningful and lasting liver disease modification.

## 3. What Constitutes Liver Disease Modification in MASLD?

Liver disease modification in MASLD should be understood as a sustained improvement in the pathological processes that drive disease progression and clinical outcomes. This concept extends beyond improvement in a single biochemical, imaging, or metabolic measure because these outcomes differ in their relationship to liver pathology and future clinical events [3,14]. A practical distinction can be made between biological response, hepatic response, histological or structural improvement, and clinical benefit. These outcomes are not interchangeable. Early responses may indicate biological activity, whereas histological improvement provides more direct evidence of pathological change and clinical outcomes provide the clearest evidence of benefit to patients [3,14].

### 3.1. Biological and Hepatic Responses

Metabolic and biochemical changes may provide early evidence of response, but their clinical significance depends on the measure evaluated. Changes in insulin resistance, circulating lipids, inflammatory markers, or aminotransferases may accompany improvement in MASLD without directly establishing improvement in steatohepatitis or fibrosis [3,9]. Alanine aminotransferase (ALT) illustrates this distinction. In patients with biopsy-proven steatohepatitis undergoing lifestyle intervention, ALT normalization was independently associated with fibrosis improvement after one year [6]. However, this association does not make ALT normalization equivalent to fibrosis regression because serum ALT levels do not directly assess liver fibrosis.

Measures that assess the liver more directly offer stronger evidence of hepatic response. MRI-PDFF quantifies hepatic fat, and reductions in this measure have been associated with histological response [15]. In a multicenter analysis, a relative reduction of at least 30% in magnetic resonance imaging proton density fat fraction was associated with histological response and improvement in steatosis, whereas its association with fibrosis improvement was not statistically significant [15]. A reduction in hepatic fat therefore supports improvement in steatosis. It does not, by itself, demonstrate fibrosis regression or modification of the long-term disease course because steatosis, inflammatory activity, and fibrosis are distinct pathological features that may not change in parallel [15].

Non-invasive fibrosis measures present a similar interpretive challenge. Liver stiffness measurements, serum fibrosis biomarkers, and composite scores are valuable for risk stratification and longitudinal assessment, but validated thresholds of improvement that reliably confirm treatment-induced histological regression or clinical benefit remain unavailable for many measures [3,9]. Newer markers are also being studied. One example is PRO-C3, a blood marker related to collagen production and liver fibrosis. PRO-C3 levels have been associated with fibrosis stage, and the PRO-C3-based ADAPT score has shown promising results for identifying advanced fibrosis. However, these approaches still require further validation before they can be widely used in routine clinical practice [16,17].

Oxidative stress is another important pathway in MASLD. When the balance between harmful oxygen molecules and the body’s antioxidant defenses is disturbed, it can contribute to liver cell injury, inflammation, and changes in liver fat metabolism. Mitochondrial dysfunction and lipid damage may further increase this stress [18,19]. Exercise may help reduce oxidative stress and improve antioxidant defenses, although the available evidence is not yet consistent and much of it comes from studies of NAFLD rather than MASLD specifically [20]. Changes in oxidative-stress markers should therefore be viewed as evidence of a biological response rather than direct proof of liver disease modification.

Serial assessment may nevertheless offer prognostic information. In patients with compensated cirrhosis related to nonalcoholic steatohepatitis, increases in liver stiffness and the NAFLD fibrosis score were independently associated with subsequent liver-related events. Changes in the Fibrosis 4 index (FIB-4 score) and enhanced liver fibrosis score were not significantly associated with these outcomes [21]. Individual non-invasive tests should therefore not be assumed to have equivalent prognostic value.

### 3.2. Histological and Structural Improvement

Liver biopsy with histological assessment remains the most direct method for evaluating the microscopic features of MASH, including steatosis, hepatocellular ballooning, lobular inflammation, and fibrosis [3]. Liver biopsy, however, is limited by sampling variability and variation in pathological interpretation. In paired biopsy specimens from 51 patients with nonalcoholic fatty liver disease, discordance of at least one fibrosis stage occurred in 41% of paired samples [22]. This finding illustrates the potential effect of the heterogeneous distribution of liver lesions on fibrosis staging. Its invasiveness limits its use for repeated longitudinal assessment [3]. Despite these limitations, histological improvement provides more direct evidence of pathological change than isolated biochemical or imaging responses. Resolution of MASH reflects improvement in disease activity, whereas fibrosis improvement reflects structural change. These outcomes may occur together, but they represent different aspects of disease and should be interpreted separately [3,14].

Fibrosis has particular importance because its stage is consistently associated with long-term prognosis. In a cohort of 229 patients with biopsy-proven NAFLD followed for up to 33 years, fibrosis stage was the strongest histological predictor of disease-specific mortality, whereas the NAFLD activity score (NAS) did not predict overall mortality [23]. This prognostic gradient has been confirmed across broader populations. In the study by Angulo et al., fibrosis stage was independently associated with the composite outcome of overall mortality or liver transplantation, while advanced fibrosis was independently associated with liver-related events [24]. Meta-analyses have subsequently demonstrated progressively higher all-cause and liver-related mortality with advancing fibrosis stage [25,26,27].

Fibrosis is also dynamic. In a meta-analysis of paired biopsy studies, 33.6% of patients showed fibrosis progression, 43.1% remained stable, and 22.3% showed improvement during follow-up [28]. Fibrosis improvement is therefore a biologically meaningful outcome, but its interpretation should consider baseline disease severity, duration of follow-up, and the limitations of the assessment method.

### 3.3. From Structural Improvement to Clinical Benefit

The strongest evidence of clinically meaningful liver disease modification is obtained when structural improvement is associated with better clinical outcomes. In a pooled analysis of 1135 patients with compensated cirrhosis related to nonalcoholic steatohepatitis, cirrhosis regression between baseline and week 48 was associated with fewer subsequent liver-related events. Events occurred in 1.1% of patients with regression and 7.2% of those without regression, corresponding to an adjusted hazard ratio of 0.16 (95% confidence interval 0.04 to 0.65) [21]. This finding provides important evidence linking structural improvement with clinical benefit, but its interpretation should remain specific to the studied population. The analysis involved patients with compensated cirrhosis and does not establish that every histological improvement at earlier stages of MASLD will produce a similar reduction in clinical events [21].

This distinction is critical because a prognostic association does not automatically establish a treatment effect. Higher fibrosis stage is consistently associated with mortality and liver-related events, but these natural history data do not prove that every treatment-induced reduction in fibrosis will produce a proportional reduction in clinical outcomes [25,26,27]. Histological improvement therefore provides strong evidence of favorable pathological change, particularly when fibrosis improves. Confidence that such improvement represents clinically meaningful disease modification is strengthened when the benefit is durable and accompanied by a lower risk of subsequent liver-related events [21].

### 3.4. Interpreting the Evidence for Disease Modification

For the purposes of this review, liver disease modification is interpreted as a graded concept rather than a binary outcome. Evidence becomes more compelling as improvement moves from biological activity and hepatic response to direct pathological change and clinically meaningful benefit. These levels are not interchangeable, and the conclusion drawn from an intervention should not exceed the outcome that has actually been measured [3,14].

Confidence in a disease-modifying effect is further strengthened when improvement is sustained and supported by evidence linking the measured response to subsequent clinical benefit. Intermediate endpoints nevertheless remain essential because major liver outcomes may require large populations and prolonged follow-up, particularly in noncirrhotic MASLD. However, improvement in an intermediate outcome should not be interpreted as proof of clinical benefit unless that relationship has been established in the relevant population and setting [3,14,21].

The main levels of response and their interpretation are summarized in Table 1. This framework will guide the interpretation of dietary and lifestyle interventions in the following sections. Table 1 complements the five-question framework presented in Section 10 by showing what each level of response can tell us about liver disease modification.

## 4. The Role of Weight Loss in Liver Disease Modification

### 4.1. Weight Loss as a Therapeutic Target

The therapeutic relevance of weight loss is reinforced by the consistency of the dose–response relationship observed across lifestyle studies. In patients with nonalcoholic fatty liver disease, a systematic review and meta-analysis found that greater weight loss was generally associated with greater improvements in liver enzymes and markers of disease activity. The magnitude of benefit varied according to the outcome assessed, and the available evidence was heterogeneous [29].

More intensive interventions can yield greater improvements in hepatic and metabolic outcomes than conventional lifestyle programs. In a 12-week multicenter randomized controlled trial involving 226 Chinese adults with overweight or obesity and nonalcoholic fatty liver disease, a comprehensive intensive lifestyle intervention produced greater reductions in liver fat, body mass index (BMI), and several metabolic measures than a conventional lifestyle intervention. The intervention included a low-carbohydrate, high-protein calorie-restricted diet, exercise, and frequent dietitian-led counseling [30].

The importance of weight loss extends beyond biochemical improvement. Histological studies have demonstrated a dose–response relationship between the magnitude of weight loss and improvement in steatohepatitis, with greater weight reduction associated with a higher likelihood of histological benefit [6]. In a systematic review and meta-analysis of 43 intervention studies, greater weight loss was associated with greater improvements in steatosis, inflammation, ballooning, and resolution of steatohepatitis, whereas evidence for a dose–response relationship with fibrosis was limited [31]. Weight loss is therefore an important therapeutic target, but its clinical significance depends on the hepatic outcome assessed.

Evidence from interventions producing substantial and sustained weight reduction further supports its therapeutic importance. In patients with obesity, bariatric surgery has been associated with marked histological improvement, including long-term resolution of steatohepatitis and progressive fibrosis regression [32]. However, the hepatic benefits of bariatric procedures cannot be attributed solely to reduced body weight. In a five-year controlled study, the greater improvement in NAFLD after Roux-en-Y gastric bypass than after adjustable gastric banding was primarily, but not entirely, explained by differences in weight loss [33].

Pharmacological studies provide a complementary perspective on the relationship between weight loss and liver response. In the SYNERGY-NASH trial, tirzepatide improved histological outcomes in patients with biopsy-confirmed MASH and stage F2 or F3 fibrosis while producing substantial weight loss. Nevertheless, the trial was not designed to determine the extent to which the histological benefit was mediated by weight reduction rather than by other metabolic effects of treatment. These findings therefore support a close relationship between weight loss and liver improvement but do not establish that changes in body weight alone account for the observed histological response [34].

However, the bariatric surgery and tirzepatide studies discussed above largely involved people with obesity or substantial excess weight. Their findings may not fully apply to patients with MASLD who do not have obesity.

### 4.2. Weight Loss as a Mediator of Intervention Effects

Weight loss may act as a pathway through which dietary or lifestyle interventions influence liver outcomes. However, parallel changes in body weight and liver outcomes do not, by themselves, establish mediation. Demonstrating mediation requires analytical evidence that changes in body weight account for part of the effect of an intervention on the outcome [35]. Such analyses remain limited in studies of lifestyle interventions for MASLD, in which changes in body weight and liver outcomes are more commonly reported concurrently than evaluated within formal mediation frameworks [29,36].

The available evidence nevertheless suggests that weight-related and weight-independent pathways may coexist. In a longitudinal cohort study of adults without NAFLD at baseline, moderate- to high-intensity exercise was associated with a lower risk of incident NAFLD. Formal mediation analysis indicated that part of this association was mediated by weight maintenance, whereas another component was independent of weight maintenance [36]. These findings cannot establish the same causal structure in patients with established MASLD, but they illustrate why changes in total body weight should not automatically be assumed to account for the full hepatic effect of lifestyle interventions.

Dietary studies provide a similar caution. In a 12-week randomized trial comparing ad libitum Mediterranean and low-fat diets, both dietary patterns reduced hepatic steatosis to a similar extent despite minimal and comparable weight loss between groups. These findings suggest that dietary changes may influence hepatic steatosis through pathways that are not fully captured by changes in total body weight [37]. Future trials should include formal mediation analyses to determine how much of the liver benefit is explained by weight loss and how much may occur through other pathways.

### 4.3. Weight Loss as an Imperfect Marker of Response

Total body weight is an accessible clinical measure, but it provides limited information about the biological changes accompanying lifestyle intervention. A systematic review of longitudinal studies found that reductions in visceral and subcutaneous adipose tissue were associated with reductions in hepatic steatosis [38]. Similar changes in body weight may reflect different changes in adipose tissue, hepatic fat, and lean mass, while relatively stable weight may conceal clinically relevant changes in body composition and hepatic steatosis [38].

The relationship between body weight and hepatic response is imperfect in intervention studies. A meta-analysis of randomized controlled trials found that exercise reduced intrahepatic fat even when clinically significant weight loss was not achieved, indicating that hepatic improvement can occur without major changes in total body mass [39]. More recent evidence using MRI-defined treatment response similarly found that exercise-associated reductions in liver fat were independent of clinically significant weight loss [40]. Although the mechanisms of exercise are considered elsewhere, these findings illustrate the limitation of using weight change alone as a measure of intervention success.

This limitation also matters over time because weight regain is common in clinical practice [12]. However, the extent to which changes in body weight remain coupled with changes in liver outcomes during weight maintenance or regain has not been adequately defined. Longitudinal studies should therefore assess body weight and liver outcomes together. Weight loss remains an important therapeutic target, but it should be interpreted alongside liver-specific outcomes (Figure 2).

## 5. Dietary and Lifestyle Pathways to Liver Improvement

Dietary and lifestyle interventions can influence MASLD through several intervention-specific pathways. For this section, the main pathways are energy balance and body weight, dietary composition and nutrient exposure, and physical activity and exercise. We emphasize the liver outcomes most consistently associated with each approach [3,9].

Behavioral support and adherence are shown in Figure 1 because they influence how well dietary and lifestyle interventions can be implemented and maintained. They are not treated as a separate pathway because they support each of the three pathways described below instead of representing a distinct biological mechanism.

### 5.1. Energy Restriction and Intentional Weight Reduction

Energy restriction creates a negative energy balance that can reduce body weight and adiposity. In MASLD, intentional weight reduction is associated with improvements in hepatic fat and metabolic function, although the relative contributions of reduced energy intake, changes in adiposity, and other components of multicomponent lifestyle interventions cannot always be separated [3,7].

In a randomized trial of patients with biopsy-proven nonalcoholic steatohepatitis, an intensive lifestyle intervention combining dietary modification, exercise, and behavioral strategies resulted in greater weight loss and histological improvement than structured education. The magnitude of weight reduction was correlated with improvement in the NAS, supporting a relationship between lifestyle-induced weight loss and improvement in disease activity [7].

Longer interventions have demonstrated histological benefit. In a six-month randomized trial of patients with biopsy-proven NAFLD, repeated liver biopsy showed improvement in the NAS after lifestyle modification, although histological responses did not differ significantly among the intervention strategies [8]. A systematic review and meta-analysis of 22 randomized clinical trials involving 2588 participants found that weight-loss interventions improved liver enzymes, hepatic steatosis, histological NAS, and the presence of steatohepatitis, whereas a statistically significant improvement in histological fibrosis was not demonstrated [29].

Energy restriction can reduce hepatic fat over relatively short periods. In the TREATY-FLD randomized trial, daily calorie restriction and time-restricted eating produced comparable reductions in intrahepatic triglyceride content, body weight, and several metabolic measures, reinforcing the importance of overall energy restriction rather than meal timing alone in that setting [41]. In a randomized trial involving patients with compensated cirrhosis due to MASLD, severe dietary energy restriction reduced body weight, liver fat, and iron-corrected T1 but did not significantly improve liver stiffness during the intervention period [42].

The most consistently demonstrated liver-specific response to energy restriction is therefore a reduction in hepatic steatosis. Greater and sustained weight reduction may be accompanied by improvement in steatohepatitis and, in some patients, fibrosis, but these structural outcomes should not be inferred from short-term reductions in liver fat alone [3,8,29].

### 5.2. Dietary Quality and Composition

Dietary composition may affect MASLD even when weight loss is limited. Changes in the type and proportion of dietary carbohydrates and fats can alter hepatic steatosis independently of large differences in total body weight, although the effects of dietary composition and energy intake are often difficult to separate in clinical studies [37,43].

Mediterranean-style dietary patterns illustrate this relationship. In a randomized crossover study of patients with NAFLD, a Mediterranean diet produced a greater reduction in hepatic steatosis and improved insulin sensitivity compared with a low-fat, high-carbohydrate diet, without a significant difference in weight loss between dietary periods [43]. However, the relative benefit of specific dietary patterns has not been consistent across studies. In a 12-week randomized trial, both ad libitum Mediterranean and low-fat diets reduced hepatic steatosis and liver enzymes, with no significant between-group difference in the reduction in liver fat or body weight [37]. These findings should be interpreted within the populations studied. Both trials involved patients with NAFLD, so the results may not fully generalize to all patients with MASLD, particularly those with different metabolic profiles or stages of disease.

Carbohydrate restriction can reduce hepatic fat, although its effects may be difficult to separate from concurrent changes in total energy intake and adiposity. In a randomized study of patients with NAFLD, low-carbohydrate dietary education was associated with greater reductions in hepatic fat and a higher frequency of ALT normalization than low-fat dietary education; however, concurrent differences in energy intake and abdominal adiposity limited attribution of these effects to carbohydrate restriction alone [44]. Similarly, a short-term intervention study found that carbohydrate restriction was associated with a marked reduction in liver fat and concurrent changes in lipid and carbohydrate metabolic pathways, although the contributions of altered macronutrient composition and concurrent energy restriction could not be fully separated [45].

The quality of dietary fat may influence hepatic fat accumulation. In a randomized overfeeding study, saturated and polyunsaturated fat produced different effects on liver and visceral fat accumulation despite similar weight gain, indicating that the metabolic consequences of energy excess may depend partly on dietary fat composition [46]. Specific dietary sources can likewise affect ectopic fat deposition. In a six-month randomized intervention, daily consumption of sucrose-sweetened cola increased liver fat compared with semiskim milk, diet cola, and water; visceral and skeletal muscle fat increased despite no significant between-group differences in body weight or total fat mass [47].

The most consistently demonstrated liver-specific effect of dietary quality and composition is improvement in hepatic steatosis. The studies discussed above do not establish consistent effects on fibrosis or long-term clinical outcomes; dietary compositional changes should therefore not be interpreted as evidence of structural disease modification unless those outcomes are directly assessed [37,43,45].

### 5.3. Physical Activity and Exercise

Physical activity and exercise can improve liver outcomes independently of clinically significant weight loss. The biological response involves multiple adaptations in energy metabolism and metabolic function, although individual mechanisms should not be inferred from changes in liver fat alone [40,48].

In a randomized trial of patients with NAFLD, 16 weeks of supervised moderate-intensity aerobic exercise decreased intrahepatic triglyceride content without significant changes in body weight or total body fat [48]. Similar findings have been reported with resistance training. In sedentary adults with clinically defined NAFLD, eight weeks of resistance exercise reduced liver fat and enhanced lipid oxidation and glucose control while improving insulin resistance without significant changes in body weight, visceral adipose tissue, or whole-body fat mass [49].

The benefits of aerobic exercise have been observed across different exercise volumes and intensities. In a randomized placebo-controlled trial, three eight-week aerobic exercise regimens reduced liver fat and visceral adiposity without clinically significant weight loss; however, reductions did not differ significantly among the exercise doses or intensities tested [50]. In a separate randomized clinical trial, aerobic and resistance training combined with dietary modification produced comparable reductions in hepatic fat and improvements in insulin sensitivity [51]. These findings indicate that more than one exercise modality can contribute to improvement in steatosis, although the independent contribution of exercise could not be separated from the concurrent dietary intervention.

Evidence from pooled randomized trials strengthens the conclusion that exercise can reduce hepatic fat independently of substantial weight loss. In a systematic review and meta-analysis of 14 randomized controlled trials involving 551 adults with NAFLD, exercise training increased the likelihood of achieving a ≥30% relative reduction in MRI-measured liver fat compared with control conditions, independently of clinically significant weight loss [40].

The most consistent liver-specific effect of exercise is therefore a reduction in hepatic steatosis. Exercise training has improved surrogate serum- and imaging-based markers associated with histological response, but these findings do not establish histological resolution of MASH or fibrosis regression. Direct interventional evidence for consistent effects of exercise alone on these structural outcomes is still limited [40,52].

### 5.4. Comparative Interpretation of Intervention Pathways

These interventions should not be regarded as interchangeable. Energy restriction primarily promotes negative energy balance and weight reduction; dietary composition can alter the nutrient and metabolic environment associated with hepatic lipid accumulation; and physical activity can improve hepatic steatosis through adaptations that are not fully dependent on substantial weight loss [7,43,48].

The evidentiary strength of the liver outcomes also differs. Hepatic steatosis is responsive to all three approaches, whereas improvement in steatohepatitis and fibrosis is more strongly supported in the context of sustained weight reduction, particularly with dietary and behavioral interventions. For exercise, direct evidence of independent histological benefit remains limited. These distinctions provide the basis for interpreting dietary and lifestyle interventions according to both the pathway targeted and the liver outcome measured (Table 2) [3].

## 6. Why Body Weight Does Not Fully Capture Biological Response

Total body weight measures mass, not where that mass has changed or how metabolic function has changed. Obesity is biologically heterogeneous, and differences in fat distribution, ectopic fat, and skeletal muscle may influence liver risk and treatment response beyond total body weight [38,53,54]. Differences in energy balance, insulin sensitivity, and metabolic function may further contribute to variation in obesity and its hepatic effects [3,9]. Similar weight trajectories may therefore reflect different biological changes. These differences are relevant to MASLD because changes in visceral adiposity, ectopic fat, and skeletal muscle may be associated with changes in hepatic steatosis that are not fully captured by total body weight alone [38].

Figure 3 illustrates why similar changes in body weight may be accompanied by different biological responses, depending on changes in visceral adiposity, ectopic fat, skeletal muscle, and metabolic fitness. As noted in Section 1, body weight is an accessible but incomplete measure of biological response; this section examines the specific changes that may be missed by weight alone.

### 6.1. Visceral Adiposity

Visceral adiposity is not directly represented by total body weight. Individuals with similar body weight or similar changes in body weight may differ substantially in the amount and distribution of abdominal adipose tissue. This distinction is relevant to MASLD because visceral fat has been associated with hepatic steatosis independently of simple measures of overall adiposity. In a prospective study of 304 individuals assessed with abdominal computed tomography and controlled attenuation parameter, visceral fat area was independently associated with significant hepatic steatosis, whereas BMI was not independently associated with steatosis after multivariable adjustment [53].

The relationship between visceral adiposity and more advanced liver disease has been demonstrated in biopsy-based cohorts. In 324 patients with NAFLD, computed tomography-measured visceral adipose tissue was independently associated with significant fibrosis and NASH after adjustment for relevant clinical and adiposity-related variables, including subcutaneous adipose tissue [55]. In a separate prospective cohort of 521 patients with biopsy-confirmed NAFLD, computed tomography-assessed visceral adiposity showed an independent association with significant fibrosis after multivariable adjustment [54]. These findings do not establish that visceral fat alone determines disease progression, but they demonstrate that total body weight does not capture all adiposity-related information relevant to liver disease severity.

Longitudinal evidence further supports the importance of changes in visceral adiposity. In an early longitudinal study of patients with NAFLD, change in visceral fat thickness showed the strongest relationship with change in hepatic steatosis in multivariable analysis, although the longitudinal sample was small [56]. More recently, a systematic review and meta-analysis of longitudinal studies found that changes in visceral adipose tissue were positively associated with changes in hepatic steatosis. The quantitative evidence was limited to a small number of studies, but the pooled correlations consistently indicated that changes in visceral fat and liver steatosis tend to occur together [38].

These observations have an important implication for interpreting body weight change. Similar reductions in total body weight may be accompanied by different reductions in visceral adiposity and therefore by different changes in hepatic steatosis or other metabolic abnormalities. Conversely, body weight may provide an incomplete description of an adverse trajectory when visceral adiposity increases disproportionately. Total body weight should therefore be regarded as an overall measure of mass rather than a direct measure of the abdominal fat distribution relevant to hepatic and metabolic abnormalities in MASLD [38,53,57].

### 6.2. Ectopic Fat

Total body weight does not directly measure ectopic fat. In MASLD, hepatic steatosis is the most clinically relevant ectopic lipid compartment, and changes in liver fat need not occur in direct proportion to changes in total body mass. In a longitudinal study of individuals with NAFLD undergoing lifestyle intervention, similar weight loss was associated with substantial interindividual variability in the reduction in liver fat [58].

The temporal pattern of metabolic change may differ from that of total body mass. In a clinical study of severe energy restriction, intrahepatic triglyceride content decreased by approximately 30% within the first week, together with marked improvement in hepatic insulin sensitivity [59]. Although this study was conducted in individuals with type 2 diabetes rather than MASLD, it indicates that substantial changes in hepatic lipid content can occur rapidly during negative energy balance and may not be fully characterized by changes in total body weight alone.

This discrepancy is evident in intervention studies in MASLD. In a randomized trial, aerobic exercise reduced intrahepatic triglyceride content without significant changes in total body weight or body fat, demonstrating that hepatic fat can change despite little alteration in overall body mass [48]. Thus, similar weight changes do not necessarily reflect similar changes in ectopic fat, making direct assessment of hepatic steatosis important. This distinction is particularly important in MASLD because hepatic fat is a defining feature of the disease [48,58].

### 6.3. Skeletal Muscle

Total body weight does not distinguish between changes in adipose and lean tissue. Similar reductions in body weight may therefore represent different combinations of fat and skeletal-muscle loss, which may have different implications for metabolic status. Longitudinal studies support the relevance of skeletal muscle to liver disease: higher relative skeletal muscle mass and increases in relative muscle mass over time were associated with a lower risk of incident NAFLD and a greater likelihood of resolution of baseline NAFLD in a large 7-year cohort [60].

Evidence indicates that skeletal-muscle loss may accompany established liver disease. In a longitudinal cohort of 52,815 adults, participants with NAFLD had a greater 5-year decline in appendicular skeletal muscle mass than those without NAFLD, and muscle loss was more pronounced in participants with higher non-invasive fibrosis scores [61]. These observational findings do not establish a causal effect of muscle loss on liver disease progression. Nonetheless, they indicate that changes in muscle and liver status may evolve in parallel.

Muscle quantity and quality may yield information not captured by total body weight. In a prospective cohort of 521 patients with biopsy-confirmed NAFLD, low skeletal muscle mass and myosteatosis, assessed by computed tomography, were independently associated with significant fibrosis after multivariable adjustment [54]. Thus, weight loss alone cannot show how much mass was lost from adipose tissue versus skeletal muscle. Total body weight should therefore be interpreted as a measure of overall mass rather than a direct indicator of the body composition changes that may accompany different biological trajectories in MASLD.

### 6.4. Metabolic Fitness

Metabolic fitness provides a further explanation for discordance between body weight and biological response. Body mass does not directly reflect functional capacity, insulin sensitivity, or cardiorespiratory fitness, and changes in these characteristics may occur independently of changes in body weight. In patients with NAFLD, increases in physical activity have been linked to improvements in liver enzymes and metabolic indices independent of weight loss [62].

Direct assessments of cardiorespiratory fitness have been associated with liver disease severity. In patients with biopsy-confirmed NAFLD, lower peak oxygen uptake was associated with greater necroinflammatory activity and the presence of steatohepatitis, although no significant differences were observed across steatosis or fibrosis categories in this small cross-sectional study [63].

The broader clinical relevance of cardiorespiratory fitness is supported by observational evidence. In the HUNT Study, low estimated cardiorespiratory fitness was strongly associated with a higher likelihood of NAFLD in cross-sectional analyses. Among participants with NAFLD, low fitness was associated with higher all-cause mortality during follow-up, independently of sedentary behavior and adherence to physical-activity guidelines; however, the observational design does not establish that improving fitness would reduce mortality [64].

Similar weight trajectories may therefore reflect different changes in body composition and metabolic function. Weight change should be interpreted alongside the specific biological outcome under evaluation rather than as a complete surrogate of response [38,54,64].

## 7. The Gut–Liver Axis in Lifestyle-Induced Liver Response

The gut microbiome may contribute to differences in liver response to dietary and lifestyle interventions, but its role requires careful causal interpretation. Microbial characteristics may change in response to an intervention, modify the magnitude of its effect, or predict a subsequent outcome without necessarily mediating the intervention effect. These possibilities represent distinct causal roles and should not be treated as interchangeable. Current evidence in MASLD therefore supports viewing the microbiome as a potential mediator, effect modifier, or biomarker rather than as an established therapeutic pathway [65,66]. Figure 4 summarizes these three possible roles.

### 7.1. The Microbiome as a Potential Mediator

The microbiome would act as a mediator if a dietary or lifestyle intervention altered the microbial ecosystem and this change accounted for part of the subsequent effect on the liver. Evidence for this causal pathway remains limited. Randomized studies have shown that dietary and exercise interventions can alter gut microbial characteristics while improving hepatic steatosis, but the concurrent occurrence of these changes does not establish that microbiome alteration mediated the hepatic response [66].

In an 8.6-month four-arm randomized trial of patients with NAFLD and prediabetes, aerobic exercise, dietary intervention, and their combination reduced hepatic fat and were associated with changes in gut microbial network characteristics. However, the study did not formally quantify the extent to which microbiome changes mediated the intervention effects on liver fat [66]. The observed hepatic improvements could have resulted through non-microbial pathways affected by the interventions.

A PREDIMED-Plus trial subanalysis of 297 participants found that changes in gut microbiota over one year of lifestyle intervention were associated with changes in the Hepatic Steatosis Index and FIB-4 score. Participants with more favorable changes in the Hepatic Steatosis Index had greater reductions in body weight and waist circumference, making it difficult to distinguish a potential microbiome-mediated pathway from the broader effects of lifestyle modification and changes in adiposity [67].

Evidence from direct microbiome-targeting interventions cautions against assuming that microbiome modification necessarily produces liver improvement. In a randomized pilot trial of patients with NAFLD and stable body weight, prebiotic treatment increased Bifidobacterium abundance but did not significantly reduce liver fat or favorably affect hepatic, metabolic, or inflammatory measures [68]. Similarly, in a double-blind randomized trial of patients with MASLD, three consecutive fecal microbiota transplantations did not significantly improve hepatic steatosis, liver biochemistry, or glucose tolerance compared with autologous transplantation, and no microbiota signature was associated with treatment response [69].

The current evidence therefore supports the microbiome as a potential mediator, but not as an established explanation for the hepatic benefits of lifestyle interventions [66,67,68]. Demonstrating mediation will require appropriately designed longitudinal studies with temporal ordering of intervention exposure, microbial change, and liver outcomes, together with formal mediation analyses that address the assumptions required for causal interpretation [35].

### 7.2. The Microbiome as an Effect Modifier

The microbiome may instead modify the effect of a lifestyle intervention on liver outcomes. In this role, differences in baseline microbial characteristics would be associated with differences in the magnitude or direction of the response to the same dietary or exercise exposure. The microbiome would therefore function as an effect modifier rather than as an intermediate on the causal pathway between the intervention and its outcome.

This possibility was explored in the randomized trial by Cheng et al. In exploratory analyses, baseline gut microbial network characteristics predicted individual reductions in liver fat following aerobic exercise. They also distinguished responders from low responders and nonresponders more effectively than age, body weight, or BMI. These analyses were performed in a small metagenomic subset and were not externally validated; therefore, they provide preliminary evidence that baseline microbial characteristics may help explain heterogeneity in lifestyle response rather than definitive evidence of effect modification [66].

A separate prospective study of a calorie-restricted diet found that baseline intestinal microbial profiles predicted clinically significant weight loss and differed between participants with and without reduction in hepatic steatosis. Predictive performance was substantially stronger for weight loss than for improvement in hepatic steatosis, indicating that the value of baseline microbiome profiles may depend on the outcome examined [70].

More recently, a small prospective study of pediatric MASLD found that baseline gut microbial profiles differed between participants who did and did not achieve predefined improvements in body weight, liver enzymes, or MRI-measured hepatic steatosis after a 12-week lifestyle intervention. These findings provide further preliminary evidence that baseline microbial characteristics may be associated with heterogeneity in lifestyle response. However, the single-arm design, small sample size, and pediatric population limit causal interpretation and generalizability to adult MASLD [71]. Baseline microbial characteristics may help explain variation in response to similar lifestyle interventions, but this remains an emerging hypothesis rather than a clinically established basis for treatment personalization.

### 7.3. The Microbiome as a Biomarker

The microbiome can also function as a biomarker without directly influencing disease progression or intervention response. A microbial signature may reflect the biological state of the host, help identify disease severity, or predict subsequent liver outcomes without itself being a causal determinant.

This is conceptually consistent with microbiome biomarker research; for example, Loomba et al. developed a fecal metagenomic signature to distinguish advanced from mild or moderate fibrosis in biopsy-proven NAFLD, demonstrating diagnostic utility without establishing causality [72].

In a prospective study of 86 patients with biopsy-proven NAFLD, Loomba et al. identified a metagenomic signature incorporating bacterial species, microbial diversity, age, and BMI that distinguished advanced fibrosis from mild or moderate fibrosis with high diagnostic accuracy. In an independent cohort, however, Lang et al. found that adding microbial features provided only marginal improvement over clinical models, while transient elastography showed the highest diagnostic performance. Together, these findings illustrate both the potential and the current limitations of microbiome-based biomarkers for advanced fibrosis [72,73].

Prospective population-based evidence supports a potential prognostic role. In a nested case–control study within a community-based cohort, baseline stool metagenomic and serum metabolomic features predicted incident NAFLD and liver fat accumulation over 4.6 years, outperforming models based on the available clinical predictors alone. These findings indicate that microbiome-related signatures may provide information relevant to future hepatic risk, although predictive performance does not establish that the identified microbial features cause disease [74].

In lifestyle-intervention research, a baseline microbial profile that predicts treatment response may function as a biomarker of likely response while also generating the hypothesis that baseline microbial characteristics modify intervention effects. Prediction alone, however, cannot establish effect modification or determine whether the microbiome causally influences treatment efficacy rather than reflecting other biological characteristics associated with response. This distinction is particularly important when interpreting machine learning models, which may identify useful predictive signatures without resolving the underlying causal mechanism [66,74].

Overall, the microbiome may act as a mediator, effect modifier, or biomarker, but evidence for causal mediation or clinically relevant effect modification remains limited. Future studies should prioritize longitudinal designs that distinguish causal microbial changes from those that accompany intervention or disease progression. Current guidelines do not support routine microbiome-directed therapy for MASLD because evidence for clinically meaningful liver benefit remains insufficient [3,75]. These distinctions fit the five-question framework in Section 10 by separating the role of the microbiome, the pathway involved, the outcome assessed, and the level of evidence needed to support liver disease modification.

## 8. Heterogeneity and Sustainability: Why Real-World Responses Differ

Lifestyle interventions do not produce uniform outcomes. Differences in disease and metabolic phenotype may influence the response achievable with a given intervention. Differences in intervention exposure and long-term sustainability may then determine whether that response is realized and maintained. These sources of variation should be distinguished because an unfavorable outcome after lifestyle intervention does not necessarily have a single explanation [76,77].

### 8.1. Biological Heterogeneity

MASLD encompasses heterogeneous disease states and cardiometabolic phenotypes. Consequently, the same lifestyle intervention may not produce identical liver outcomes in every individual, even when the intervention is broadly comparable. The relationship between weight reduction and hepatic improvement may vary according to the baseline phenotype and the outcome assessed [6,76].

This heterogeneity was illustrated in a 48-week observational cohort of 397 patients with MASLD undergoing lifestyle modification. Achievement of predefined weight-loss targets did not consistently correspond to MRI-PDFF response: 24.8% of participants who achieved the target showed no reduction in liver fat, whereas 29.0% of those who did not achieve the target nevertheless showed a reduction. Baseline steatosis, circulating free fatty acids, and physical activity were among the characteristics associated with these discordant responses, indicating that weight loss alone did not explain all variation in hepatic response [78].

Differences in metabolic phenotype may further influence the amount of weight reduction associated with improvement. In a randomized lifestyle intervention, approximately half of non-obese participants achieved NAFLD remission with 3–5% weight loss, whereas a similar proportion of obese participants required 7–10% weight loss. Among non-obese participants, lower baseline intrahepatic triglyceride content was independently associated with remission [76]. These findings suggest that the relationship between weight reduction and hepatic improvement does not conform to a single threshold across clinical phenotypes.

Baseline characteristics may be associated with response, although their predictive value remains uncertain. In a prospective longitudinal study of 108 patients with biopsy-proven NAFLD who underwent paired liver biopsy and Magnetic Resonance Imaging-Proton Density Fat Fraction (MRI-PDFF) assessment, routinely measured baseline characteristics did not distinguish participants with histological response from those without response [79]. In contrast, a prospective MASLD cohort undergoing a 6-month behavioral intervention identified associations between baseline anthropometric and metabolic characteristics and the magnitude of change in hepatic steatosis [80]. These findings show that baseline features may contribute to response heterogeneity, but no established baseline profile reliably predicts individual response across populations and outcomes.

### 8.2. Exposure Heterogeneity

A prescribed lifestyle intervention is not necessarily the intervention actually received. Participants may differ in the extent to which dietary and physical-activity targets are achieved, their engagement with behavioral support, and the duration of participation. Variation in these factors can produce different levels of intervention exposure and may therefore contribute to differences in observed outcomes [77].

The 52-week prospective study by Vilar-Gomez et al. illustrates this principle. Among 293 patients with biopsy-proven NASH encouraged to undertake lifestyle modification, only 88 participants achieved at least 5% weight loss. The extent of weight loss was independently associated with histological improvement, and the highest rates of steatohepatitis resolution and fibrosis regression occurred among participants who lost at least 10% of body weight. Thus, differences in the extent to which lifestyle changes are achieved can contribute substantially to variation in liver outcomes [6].

Program exposure may depend on the duration and delivery of the intervention. In a structured lifestyle program involving adults with metabolic syndrome, longer program participation was the strongest independent predictor of achieving at least 5% weight loss [81]. In a separate 12-month randomized trial in adults with NAFLD, a dietitian-led community-based lifestyle program produced greater reductions in intrahepatic triglyceride content and body weight and higher rates of NAFLD remission than usual care [82]. Although these studies did not isolate the independent contribution of each component of intervention exposure, they show that differences in participation and program delivery can influence achieved outcomes.

The distinction between biological heterogeneity and insufficient intervention exposure is therefore essential. A participant with limited dietary or physical-activity change, poor engagement, or early discontinuation cannot be interpreted in the same way as a participant with substantial exposure to the intended intervention who does not improve. Failure to distinguish these situations may lead to inadequate exposure being interpreted as apparent biological non-response [83].

### 8.3. Sustainability and Long-Term Effectiveness

Initial improvement does not guarantee durable effectiveness. For lifestyle interventions to provide sustained benefit, the required behavioral changes must remain feasible and be maintained beyond the period of intensive support. The relevant question is therefore not only whether an intervention can improve liver-related outcomes, but whether its benefits persist as the intervention is incorporated into everyday life [3].

Long-term evidence on the durability of lifestyle-related liver benefits in MASLD remains limited. In a 2-year randomized trial of 98 adults with overweight or obesity and NAFLD, personalized energy-restricted dietary interventions produced their greatest weight loss at 6 months, followed by partial weight regain at 12 and 24 months. Nevertheless, metabolic and hepatic improvements were maintained during follow-up, suggesting that partial weight regain does not necessarily result in immediate loss of all biological benefit [84].

The available evidence remains insufficient to establish whether these benefits are sustained over longer periods or translate into long-term clinical benefit. The VITALISE feasibility study, which included 35 participants, was designed to assess feasibility over 6 months rather than long-term effectiveness [85]. Larger studies with longer follow-up are therefore needed to determine the durability of lifestyle-related liver improvements. Lifestyle response should be considered in relation to phenotype, achieved intervention exposure, and sustainability rather than reduced to a simple responder–non-responder classification.

## 9. From Short-Term Response to Causal Evidence of Liver Disease Modification

Lifestyle interventions can favorably affect biological and intermediate liver outcomes, but the evidentiary strength of such effects depends on the outcome measured, duration of follow-up, and study design. Improvements in steatosis, biochemical markers, histology, or non-invasive measures should therefore be distinguished from evidence of reduced cirrhosis, hepatic decompensation, HCC, or liver-related mortality [3].

### 9.1. Short-Term Liver Markers and Surrogate Endpoints

Lifestyle studies commonly evaluate hepatic fat, aminotransferases, liver stiffness, non-invasive fibrosis tests, or histological features. These outcomes reflect different biological domains and should not be interpreted as interchangeable measures of disease modification. MRI-PDFF provides a quantitative measure of liver fat, and changes in MRI-PDFF have been associated with histological response in MASH. However, a reduction in liver fat should be interpreted as evidence of biological response rather than as direct evidence of reduced long-term liver-related events [86].

A similar distinction applies to non-invasive markers of fibrosis. Liver stiffness measurements and serum-based fibrosis tests can provide prognostic information, but changes in these measures do not automatically establish histological fibrosis regression. Interpretation is further complicated by within-person measurement variability and uncertainty about what magnitude of change represents a biologically meaningful response [87].

Histological endpoints provide stronger evidence of change in liver pathology but remain intermediate rather than clinical outcomes. For resmetirom, the U.S. Food and Drug Administration (FDA) granted accelerated approval using histological endpoints considered reasonably likely to predict clinical benefit: resolution of MASH without worsening of fibrosis and improvement in fibrosis without worsening of MASH [88]. Regulatory use of these endpoints does not establish that every intervention producing improvement in them will reduce future liver-related events.

### 9.2. What Randomized Lifestyle Trials Establish

Randomized trials provide the strongest basis for estimating the causal effects of defined intervention strategies on the outcomes measured during follow-up. However, the resulting causal inference remains limited to the intervention strategy, study population, duration, and endpoints actually examined [89].

This principle is directly relevant to lifestyle intervention research. Randomized trials have shown that structured dietary and behavioral interventions can improve hepatic steatosis and related metabolic outcomes over several months to two years. However, they have generally not been designed or powered to detect differences in cirrhosis, hepatic decompensation, HCC, or liver-related mortality. In the 18-month DIRECT-PLUS trial, a green-Mediterranean dietary intervention resulted in greater reductions in intrahepatic fat than Mediterranean dietary intervention and healthy dietary guidelines, while all groups were encouraged to undertake physical activity [90]. The trial therefore provides experimental evidence of an intervention effect on hepatic steatosis rather than direct evidence of reduced long-term liver-related events.

Likewise, randomized lifestyle studies demonstrating histological or imaging improvement establish causal effects on the outcomes measured during the study but do not by themselves establish persistence beyond the intervention period or long-term clinical benefit. Causal conclusions should therefore remain proportionate to the outcomes and duration actually evaluated [89].

A further issue concerns adherence. The intention-to-treat effect estimates the effect of assignment to an intervention strategy, whereas the effect of sustained adherence represents a different causal estimand. Subsequent adherence and behavioral exposure occur after randomization and may be influenced by prognostic factors. Analyses based on achieved adherence or weight loss therefore do not retain the same protection against bias as the randomized comparison of assigned interventions and require additional assumptions and appropriate methods [91,92].

### 9.3. What Observational Evidence Adds and Cannot Resolve

Observational studies can provide longer follow-up and evaluate outcomes that are difficult to capture in conventional lifestyle trials. Longitudinal cohorts can therefore examine whether changes in body weight, physical activity, diet, or metabolic status are associated with subsequent hepatic or other long-term clinical outcomes [93,94].

However, longer follow-up does not eliminate confounding. Individuals who sustain healthier behaviors may differ systematically from those who do not in disease severity, comorbidity, socioeconomic circumstances, healthcare access, or other factors related to both behavior and prognosis. Consequently, an association between sustained lifestyle behavior and a favorable long-term outcome cannot automatically be interpreted as a causal effect of the behavior itself [95].

Time-varying exposure creates an additional challenge. Weight, physical activity, diet, metabolic status, and liver disease severity may all change during follow-up. Earlier lifestyle behavior may influence subsequent metabolic status, which may then affect both later behavior and the risk of clinical outcomes. Conventional regression approaches can therefore produce biased estimates when time-varying confounders are affected by previous exposure and influence subsequent exposure and outcome. Estimating the effects of sustained lifestyle strategies under these conditions may require causal methods designed to address time-varying confounding, including marginal structural models and other g-methods [96]. Thus, observational evidence complements randomized evidence by extending follow-up to clinically important outcomes and routine settings. Causal interpretation, however, depends on assumptions about confounding and other sources of bias that randomization is designed to minimize.

### 9.4. Target-Trial Emulation and the Causal Gap

Target-trial emulation offers a framework for specifying the causal question, treatment strategies, follow-up, outcomes, and analysis before examining observational data. It can strengthen the design and transparency of observational causal analyses, but it does not remove the need for assumptions about confounding, treatment assignment, measurement, and follow-up [97]. Explicit specification of the target trial can help prevent design errors, particularly when eligibility, treatment assignment, and the start of follow-up are misaligned [98].

This framework could address an important gap in MASLD research. A relevant long-term causal question is whether sustained adherence to a clearly defined lifestyle strategy lowers the risk of cirrhosis or other liver-related events. Conventional randomized trials addressing such questions are challenging. Sustained behavioral strategies require prolonged implementation and follow-up, while infrequent clinical outcomes make such studies resource-intensive and operationally complex. Well-characterized longitudinal data may therefore provide an opportunity to estimate the effects of sustained lifestyle strategies when the intervention strategies, exposure timing, confounders, and outcomes are adequately defined and measured [89,98].

Target-trial emulation is nevertheless not a substitute for randomization. Because treatment is not randomly assigned, causal identification depends on assumptions regarding confounding, measurement, follow-up, and the specification of intervention strategies [98]. The TARGET Statement recommends transparent reporting of the causal question, target-trial protocol, identifying assumptions, the mapping of the protocol to the available data, and sensitivity analyses addressing the influence of design and analytic choices [97].

### 9.5. What Would Demonstrate Durable Disease Modification?

The strength of the claim should match the outcome measured: liver fat reduction indicates biological response, histological improvement indicates pathological change, and sustained reductions in liver-related clinical events provide the strongest evidence of modification of the clinical course [15,88].

No single study design is likely to resolve every component of this question. Randomized trials are best suited to establishing causal effects of defined intervention strategies on outcomes measured during follow-up. Long-term observational studies can extend the evidence to clinical outcomes and real-world durability, whereas target-trial emulation can strengthen causal inference by explicitly defining the intervention strategies, time zero, confounding structure, and identifying assumptions [97,98]. The complementary contribution and limitations of different evidence approaches are summarized in Table 3.

Overall, lifestyle interventions can cause meaningful short-term improvements in liver-related biological and intermediate outcomes, whereas evidence for durable causal effects on major liver-related events remains limited [15,90].

## 10. A Framework for Evaluating Lifestyle-Induced Liver Disease Modification

Lifestyle-induced liver disease modification cannot be established simply by asking whether a lifestyle intervention “works.” A more informative evaluation requires five linked questions: Who? What? Through which pathway? What outcome? For how long and with what evidence? These questions provide a framework for determining whether an observed response represents a short-term biological change or supports a broader claim of meaningful and durable modification of MASLD (Figure 5).

Who? The starting point is the patient phenotype and disease stage. MASLD encompasses a spectrum ranging from steatosis and MASH to varying stages of fibrosis and cirrhosis, with HCC as a potential complication. The expected effects of a lifestyle strategy should therefore be interpreted in relation to the population and disease stage in which it is applied rather than assumed to be uniform across the MASLD spectrum. This is particularly important in advanced disease, where dietary and lifestyle recommendations may need to be adapted according to liver disease severity, nutritional status, and the presence of sarcopenia or sarcopenic obesity [3].

What? The intervention must be defined as a specific strategy rather than described simply as “lifestyle modification.” Dietary change, physical activity, and behavioral support can differ in content, intensity, duration, mode of delivery, and feasibility. Weight change should be interpreted according to its role within the intervention pathway rather than automatically treated as the intervention itself. Meaningful evaluation therefore requires clarity about the intended strategy, the behavioral exposure actually achieved, and whether that exposure can be maintained over time [3,77].

Through which pathway? The proposed biological pathway should connect the intervention to the expected response. Depending on the strategy, relevant pathways may involve changes in energy balance, adiposity, insulin sensitivity, hepatic and systemic substrate metabolism, physical fitness, and other metabolic processes that influence hepatic steatosis and liver injury. Mechanistic plausibility, however, should not be confused with evidence of disease modification. The pathway explains how an effect may occur, whereas the outcome determines what has actually been demonstrated [3,9].

What outcome? The endpoint must be proportionate to the claim. Improvement in liver fat, aminotransferases, non-invasive measures, histology, or clinical events provides different information and should not be treated as equivalent evidence. A biological response may show that an intervention affected a relevant process. Histological improvement provides stronger evidence of change in liver pathology, while reductions in liver-related clinical events provide the most direct evidence of modification of the clinical disease course. Treatment response should therefore be interpreted according to the clinical relevance of the endpoint and the strength of evidence supporting it [99].

For how long and with what evidence? The evidence must be judged by whether follow-up is long enough for the outcome of interest and whether the study design can support the relevant causal claim. Randomized trials provide causal evidence for outcomes measured during follow-up, whereas observational studies can extend assessment to longer-term clinical outcomes but remain vulnerable to confounding. Target-trial emulation can strengthen the causal analysis of longitudinal observational data but does not replace randomization [89,97,98]. Long-term evidence that lifestyle interventions reduce major liver-related outcomes remains limited [3].

These dimensions should be considered together because the strength of a disease-modification claim depends on their alignment. Together, they provide a structured basis for distinguishing biological improvement, pathological change, and meaningful and durable modification of liver disease.

## 11. Conclusions

Dietary and lifestyle interventions can produce meaningful improvements in MASLD, particularly reductions in hepatic steatosis. Greater weight loss is associated with greater histological improvement. Weight loss alone does not fully capture treatment response. Exercise, diet quality, body composition, and physical fitness may all contribute to improvements in liver health. The five-question framework helps interpret these effects by asking who was treated, what intervention was delivered, through which pathway it may act, what outcome was measured, and for how long and with what evidence. Randomized trials provide causal evidence for outcomes measured during follow-up, while long-term observational studies provide information on durability and remain subject to confounding. Target-trial emulation can strengthen causal assessment without replacing randomization. Overall, weight loss remains an important therapeutic target and should not be equated with liver disease modification. Claims of liver disease modification require appropriate outcomes, adequate follow-up, clearly defined intervention exposure, and study designs that support the conclusion. As a narrative review, we did not use formal systematic-review methods, perform a formal risk-of-bias assessment, or conduct a quantitative meta-analysis.

## Figures and Tables

**Figure 1:**
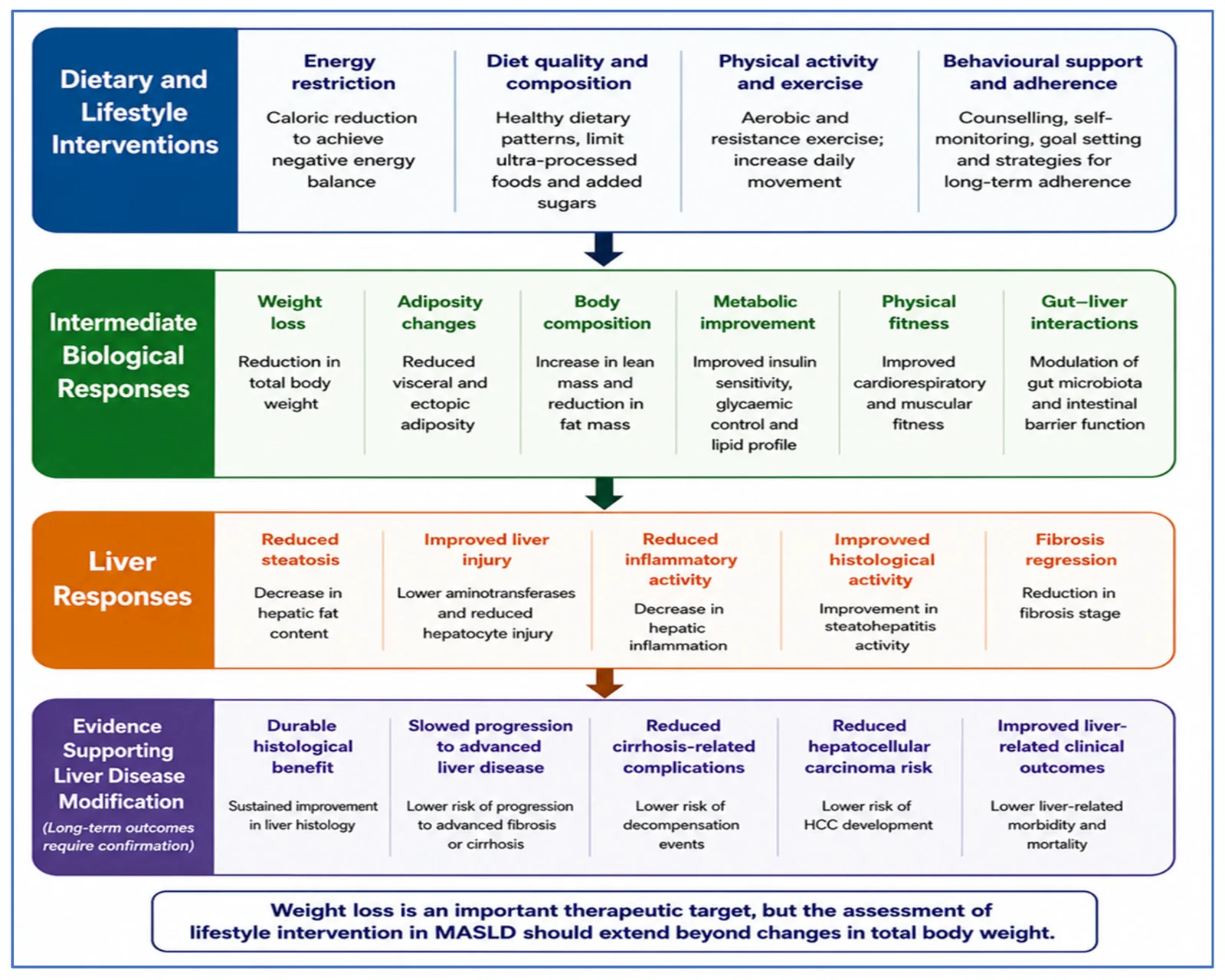
From weight loss to liver disease modification: a conceptual framework for dietary and lifestyle interventions in MASLD. Dietary and lifestyle interventions influence MASLD through weight loss and other biological responses involving adiposity, body composition, metabolic function, physical fitness, and gut–liver interactions. These changes may improve steatosis, liver injury, inflammation, histological activity, and fibrosis. Sustained histological benefit and favorable long-term clinical outcomes provide stronger evidence of liver disease modification. Weight loss is an important therapeutic target but does not by itself define treatment effectiveness in MASLD.

**Figure 2:**
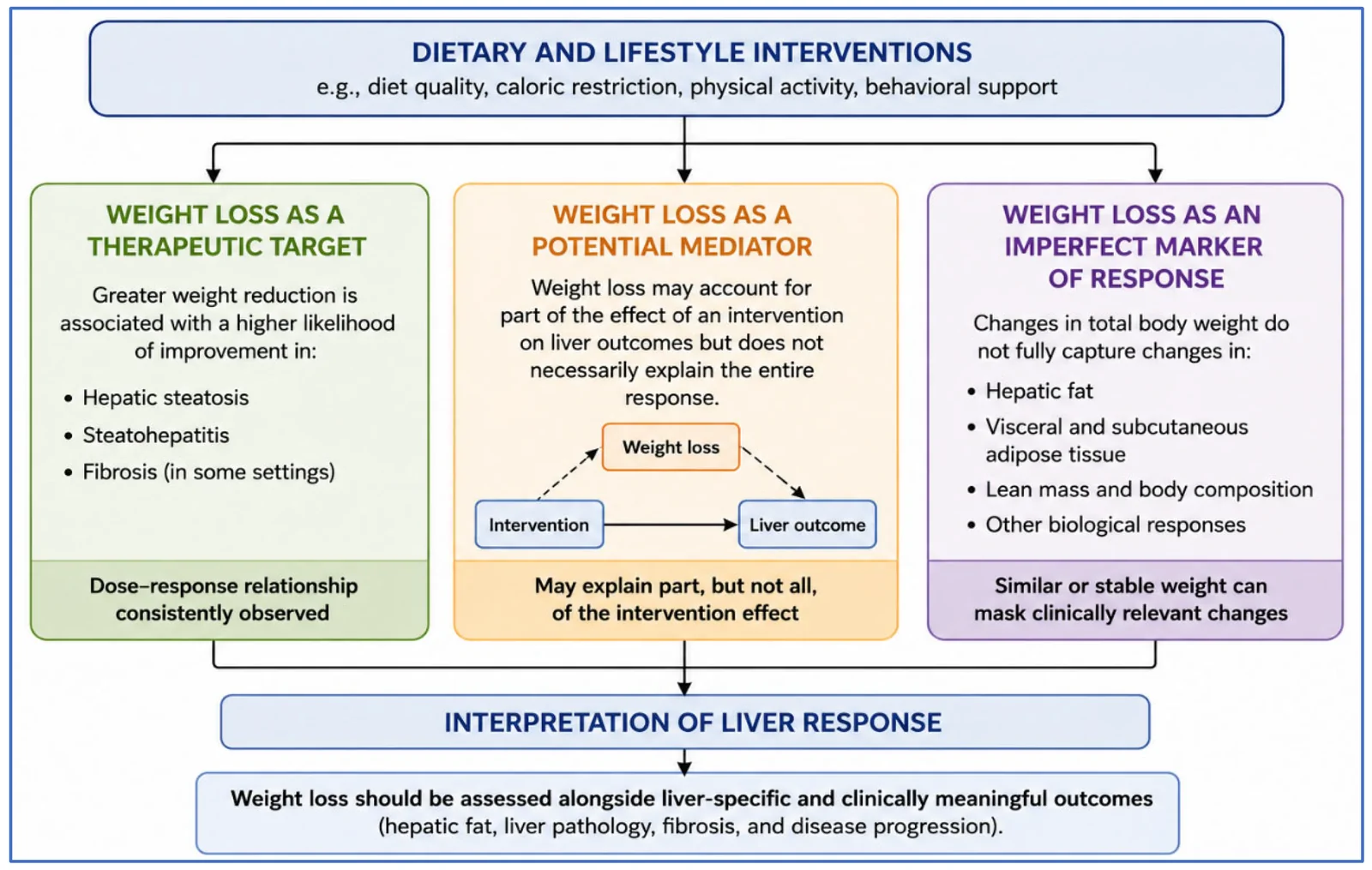
Three roles of weight loss in dietary and lifestyle interventions for MASLD. Weight loss may serve as a therapeutic target, a potential mediator of intervention effects, and an imperfect marker of response. Its interpretation should therefore consider liver-specific and clinically meaningful outcomes, including hepatic fat, liver pathology, fibrosis, and disease progression.

**Figure 3:**
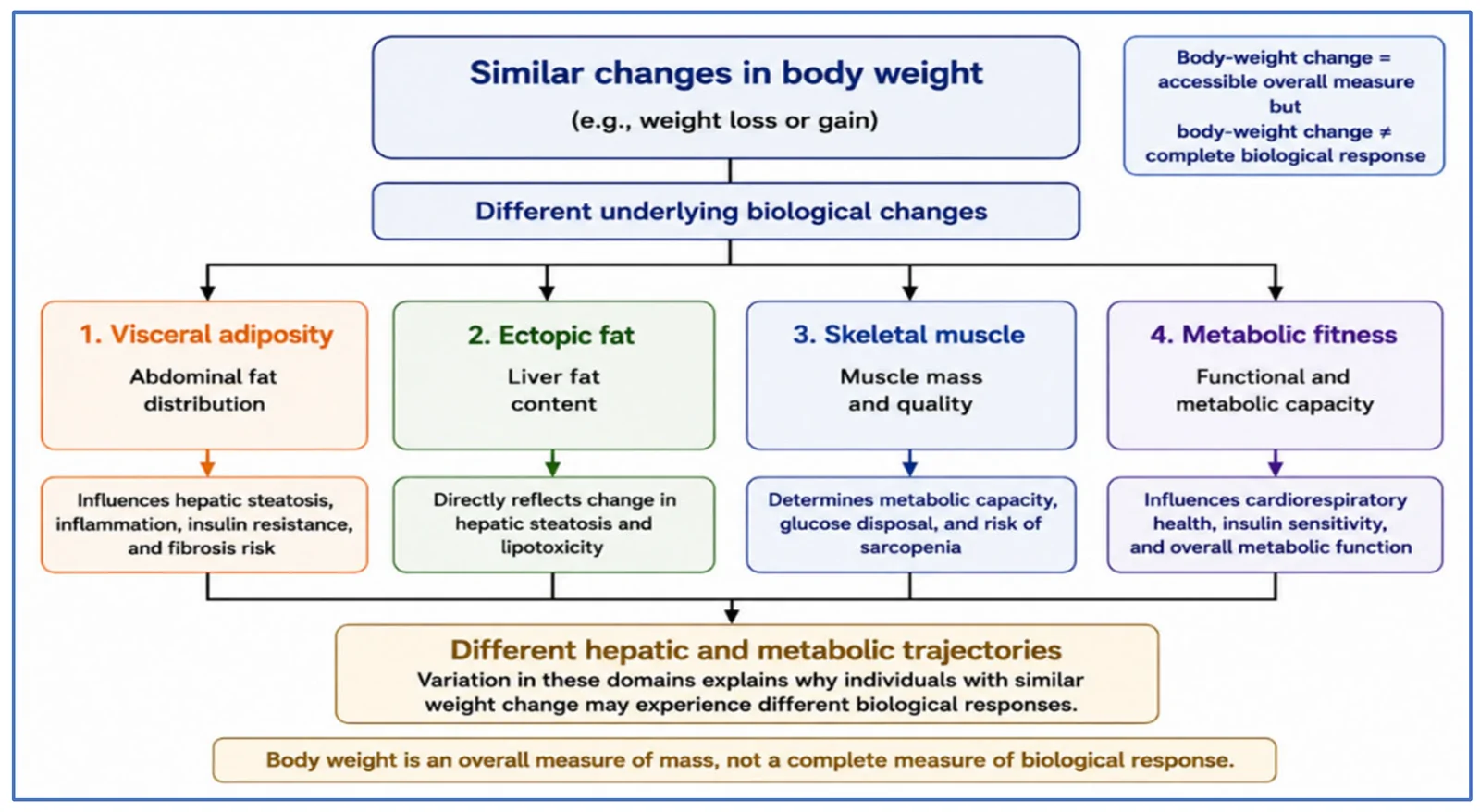
Similar changes in body weight can represent different biological responses in MASLD. Changes in visceral adiposity, ectopic fat, skeletal muscle, and metabolic fitness may differ despite similar weight trajectories, contributing to different hepatic and metabolic outcomes. Body weight therefore provides an overall measure of mass but not a complete measure of biological response.

**Figure 4:**
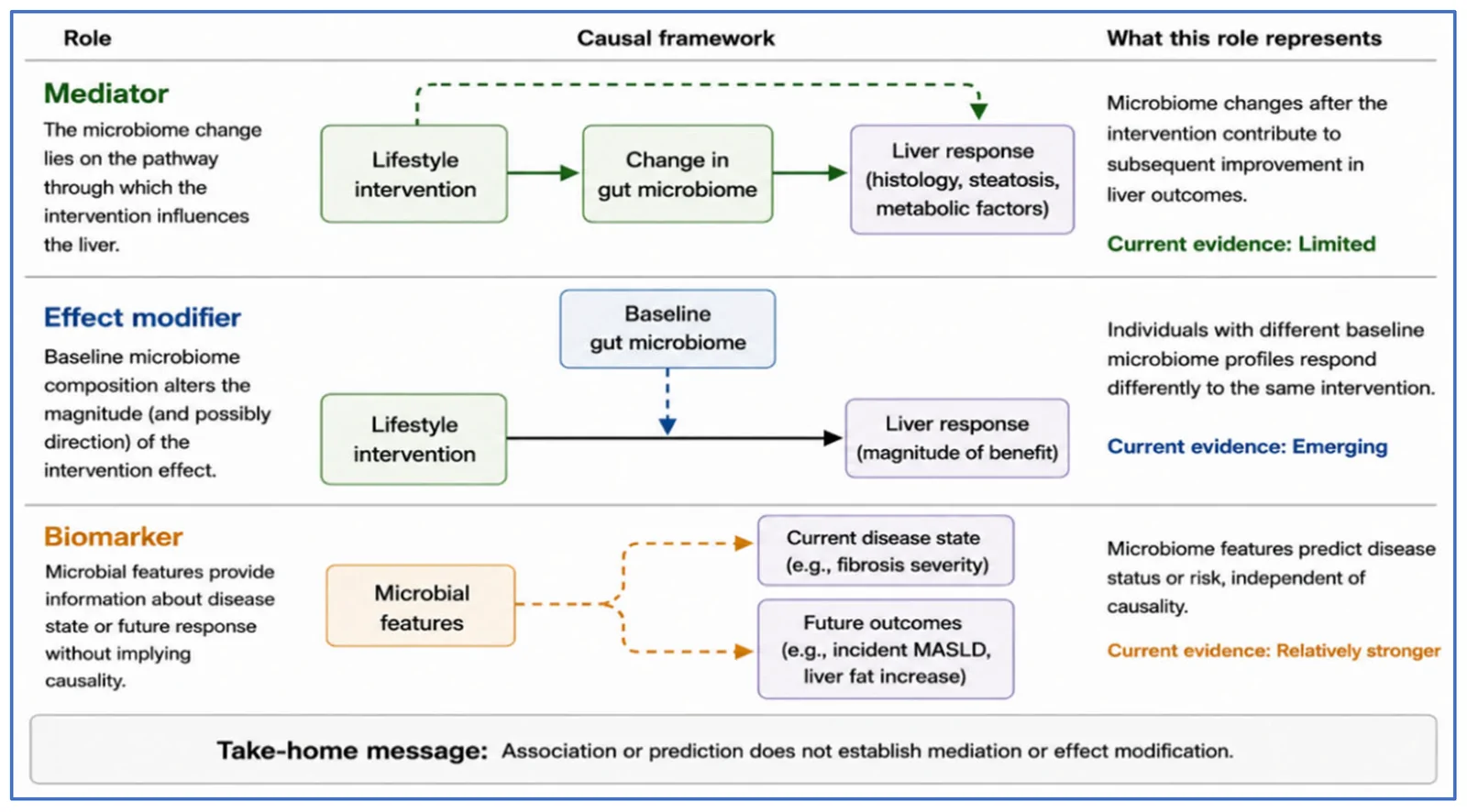
Conceptual roles of the gut microbiome in lifestyle-induced liver response. The microbiome may mediate part of the intervention effect, modify response magnitude, or serve as a biomarker of disease or treatment response. The evidence needed differs by role. For mediation, the microbial change should occur before the liver response, and formal mediation analysis is needed. Effect modification requires testing whether the intervention effect differs across groups. Biomarker value can be supported by consistent and replicated predictive performance without requiring a causal claim.

**Figure 5:**
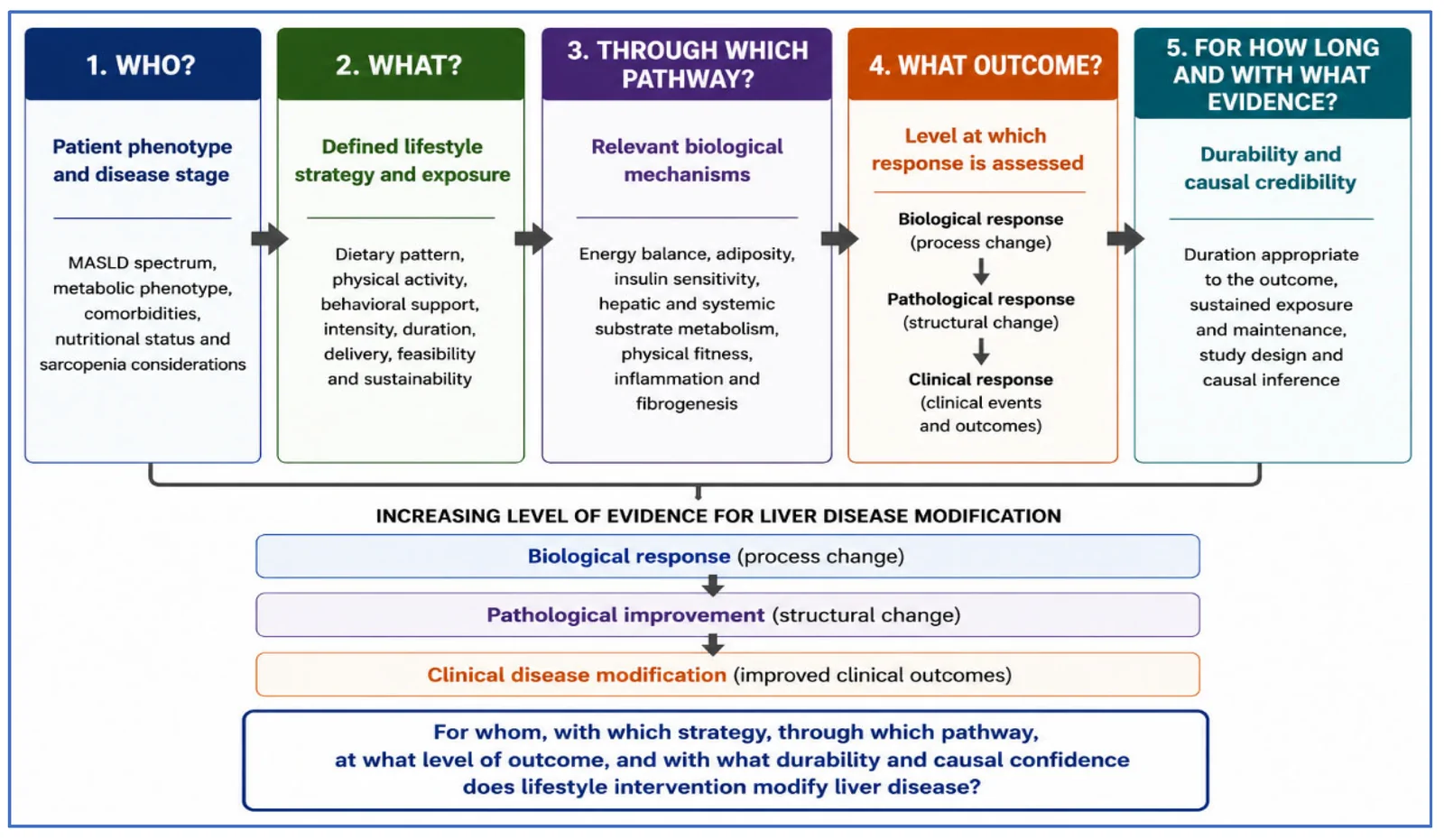
Framework for evaluating lifestyle-induced liver disease modification in MASLD. The framework links patient context, intervention strategy, biological pathway, outcome level, duration, and causal credibility to determine the strength of evidence supporting a claim of liver disease modification.

**Table 1:** Framework for interpreting evidence of liver disease modification in MASLD.

Response Level	Representative Outcomes	What the Finding Supports	Key Limitation	Refs.
Biological response	Insulin resistance, circulating lipids, inflammatory markers, aminotransferases	Biological activity relevant to MASLD	Does not directly establish improvement in liver pathology	[3,9]
Hepatic response	Liver injury markers, hepatic fat, non-invasive measures of fibrosis or disease severity	A measurable liver-related response	The relationship with histological and clinical benefit varies by measure	[3,9,15]
Histological improvement	Resolution of MASH, improvement in steatosis, ballooning, or lobular inflammation	Direct evidence of improvement in liver pathology	Requires liver biopsy and may be affected by sampling and reader variability	[3,14,22]
Structural improvement	Improvement or regression of fibrosis	Direct evidence of change in a pathological feature strongly associated with prognosis	Histological regression does not by itself prove proportional clinical benefit	[21,23,24,25,26]
Clinical benefit	Lower incidence of cirrhosis progression, hepatic decompensation, HCC, liver transplantation, or liver-related mortality	Direct evidence of clinically meaningful benefit	Requires prolonged follow-up and sufficient event accrual	[3,14,21]

**Table 2:** Principal dietary and lifestyle pathways to liver improvement in MASLD.

Intervention Approach	What Changes?	Principal Pathway	Most Consistent Liver Outcome	Key Limitation	Refs.
Energy restriction and intentional weight reduction	Energy intake, body weight, and adiposity decrease	Negative energy balance and reduced adiposity	Steatosis; histological improvement is more strongly supported with greater sustained weight loss	Energy restriction is usually part of multicomponent interventions; short-term reduction in liver fat does not establish fibrosis improvement	[7,29]
Dietary quality and composition	Carbohydrate, fat, and dietary sources are modified	Altered nutrient exposure and metabolic substrate handling	Steatosis, sometimes with limited weight change	Effects of diet composition and energy intake are often difficult to separate; structural and clinical outcomes remain insufficiently established	[37,43,46]
Physical activity and exercise	Aerobic or resistance activity is increased	Metabolic adaptations not fully dependent on substantial weight loss	Steatosis, including weight-independent reductions in liver fat	Direct evidence for consistent exercise-induced histological resolution or fibrosis regression remains limited	[40,48]

**Table 3:** Study designs and the evidence required to evaluate durable liver disease modification after lifestyle intervention in MASLD.

Evidence Approach	Main Question Addressed	What It Can Establish	Principal Limitation
Short-term biological and imaging studies	Does the intervention produce a measurable liver-related response?	Changes in liver fat, biochemical markers, or other intermediate outcomes [86]	Does not establish durable pathological or clinical benefit.
Randomized controlled trials	Does assignment to a defined intervention cause improvement in the measured outcomes?	Causal effects on outcomes observed during follow-up [89]	Causal inference is limited to the intervention, population, follow-up, and outcomes studied.
Long-term observational cohorts	Are sustained lifestyle patterns associated with subsequent clinical outcomes?	Associations with long-term clinical outcomes, including mortality, under routine conditions [93,94]	Confounding and other biases can limit causal interpretation.
Target-trial emulation	What would be the effect of a specified sustained intervention strategy?	Causal effect estimates from observational data under stated assumptions [97,98]	Cannot eliminate bias from unmeasured confounding, measurement error, or violations of identifying assumptions.

## Data Availability

No new data were created or analyzed in this study. Data sharing is not applicable to this article.
